# Sulforaphane prevents angiotensin II‐induced cardiomyopathy by activation of Nrf2 through epigenetic modification

**DOI:** 10.1111/jcmm.16504

**Published:** 2021-04-01

**Authors:** Xuling Su, Shudong Wang, Haiying Zhang, Ge Yang, Yang Bai, Pinyi Liu, Lingbin Meng, Xin Jiang, Ying Xin

**Affiliations:** ^1^ Department of Radiation Oncology The First Hospital of Jilin University Changchun China; ^2^ Key Laboratory of Pathobiology Ministry of Education Jilin University Changchun China; ^3^ The Center of Cardiovascular Diseases The First Hospital of Jilin University Changchun China; ^4^ The Center of Cardiac Surgery The First Hospital of Jilin University Changchun China; ^5^ Department of Hematology and Medical Oncology Moffitt Cancer Center Tampa FL USA

**Keywords:** angiotensin II, epigenetics, nuclear factor E2 related factor 2, oxidative stress, sulforaphane

## Abstract

Nuclear factor erythroid 2‐related factor (Nrf2) is an important regulator of cellular antioxidant defence. We previously showed that SFN prevented Ang II‐induced cardiac damage via activation of Nrf2. However, the underlying mechanism of SFN’s persistent cardiac protection remains unclear. This study aimed to explore the potential of SFN in activating cardiac Nrf2 through epigenetic mechanisms. Wild‐type mice were injected subcutaneously with Ang II, with or without SFN. Administration of chronic Ang II‐induced cardiac inflammatory factor expression, oxidative damage, fibrosis and cardiac remodelling and dysfunction, all of which were effectively improved by SFN treatment, coupled with an up‐regulation of Nrf2 and downstream genes. Bisulfite genome sequencing and chromatin immunoprecipitation (ChIP) were performed to detect the methylation level of the first 15 CpGs and histone H3 acetylation (Ac‐H3) status in the Nrf2 promoter region, respectively. The results showed that SFN reduced Ang II‐induced CpG hypermethylation and promoted Ac‐H3 accumulation in the Nrf2 promoter region, accompanied by the inhibition of global DNMT and HDAC activity, and a decreased protein expression of key DNMT and HDAC enzymes. Taken together, SFN exerts its cardioprotective effect through epigenetic modification of Nrf2, which may partially contribute to long‐term activation of cardiac Nrf2.

## 1 INTRODUCTION

The renin‐angiotensin system (RAS) exhibits a significant effect for maintenance of body electrolytes and fluid levels, and cardiovascular homeostasis. The RAS is traditionally regarded as a circulating endocrine system. It is now demonstrated that local RAS is an autocrine‐paracrine system that exists in nearly every target tissue throughout the body, including kidney,^1^ liver,^2^ brain,^3^ the blood vessels^4^ and even the heart.^5^ Cardiac local Angiotensin II (Ang II, the major effector of RAS) can be induced under various pathological conditions, such as cardiac hypertrophy,^6^ atrial fibrillation^7^ and diabetic cardiomyopathy.^8^, ^9^, ^10^ Increased cardiac Ang II stimulates inflammation, apoptosis, fibrogenesis and hypertrophy, all of which are recognized as pivotal contributors to the pathogenesis of cardiomyopathy.^11^, ^12^

Ang II has blood pressure‐dependent effects that induce hypertension, leading to left ventricular hypertrophy reflected by the accumulation of extracellular matrix and increased cardiomyocyte size.^12^ Apart from its pressor effect, the most important role of Ang II in the heart is to enhance reactive oxygen species (ROS) production independent of its haemodynamic effect.^13^, ^14^, ^15^ Mechanistically, Ang II activates its specific Ang II type 1 receptor (AT1R), then induces an ROS overproduction that dependents on NADPH, resulting in oxidative stress,^16^, ^17^ and the subsequent a series of signalling cascades activation, such as matrix metalloproteinases, the pro‐inflammatory pathway NF‐κB and mitogen‐activated protein kinase (MAPK). These signalling cascades drive myocardial hypertrophy, apoptosis and inflammation, ultimately contributing to the development of cardiomyopathy.^17^ A large number of clinical trials support the notion that exogenous ROS scavengers (such as vitamins) may cause adverse effects for patients with cardiovascular diseases (CVD). For example, long‐term consumption of vitamin E at the high‐dose results in increased risk of mortality for patients suffering from coronary heart disease and haemorrhagic stroke.^18^, ^19^ Of note, the antioxidant N‐acetyl‐cysteine and metallothionein attenuate overproduction of ROS and cardiac oxidative damage.^20^, ^21^ These studies underscore that improving the endogenous antioxidant capacity will be a prospective approach for prevention of cardiac damage caused by Ang II.

The nuclear factor erythroid 2‐related factor (Nrf2) is a major transactivator of hundreds of cytoprotective genes, which have cytoprotective functions through restoring redox balance and repairing cell damage caused by exposure to stress conditions (such as oxidative or electrophilic stress). These cytoprotective genes encoding detoxification enzymes, such as NAD(P)H:quinone reductase (NQO1), haem oxygenase 1 (HO‐1), catalase (CAT), glutamate‐cysteine ligase (GCL), glutathione S transferase (GST), UDP‐glucuronosyltransferases (UGT) and superoxide dismutase (SOD).^22^ It has been determined that Nrf2 is essential to protect against cardiomyopathy induced by Ang II.^23^, ^24^, ^25^ The overexpression of Nrf2 inhibits cardiomyopathy and ROS produced by Ang II administration, whereas the absence of Nrf2 exacerbates cardiac hypertrophy, inflammation, fibrosis and oxidative stress in cultured cardiomyocytes and Nrf2‐deficient mice.^23^, ^25^ Therefore, Nrf2 is considered to be a promising drug target for preventing heart damage caused by Ang II.

Sulforaphane (SFN) is a naturally occurring isothiocyanate compound extracted from broccoli sprouts. Reportedly SFN with multiple pharmacological activities exerts anticancer, anti‐inflammatory, antioxidant and neuroprotective effects.^26^, ^27^ With these, SFN has been promoted for general health and wellness, such as SFN enrichment in naturally consumed vegetables, the safe and efficacy of oral SFN, and the commercially available SFN supplement.^28^, ^29^ Epidemiological studies have indicated that the mortality of patients attacked by CVD is negatively related to the consumption of cruciferous vegetables.^30^ As an effective activator of Nrf2, SFN can function as an indirect antioxidant by inducing Nrf2‐mediated antioxidant signalling.^31^ Numerous studies demonstrated that SFN can effectively prevent various cardiomyopathies by activation of Nrf2, including hypoxia‐induced cardiomyopathy,^32^ doxorubicin‐induced heart failure^33^ and diabetic cardiomyopathy.^10^, ^34^, ^35^ In cultured adult cardiomyocytes, SFN protects against H_2_O_2_–induced cellular oxidative damage and reduces ROS production, accompanied by an up‐regulation of Nrf2.^36^ Additionally, it has been confirmed that SFN can inhibit cardiac hypertrophy and inflammation in Ang II‐stimulated rat H9c2 cells in vitro.^10^ These studies prove that SFN may target Nrf2 to attenuate cardiac diseases induced by oxidative stress. Moreover, our previous study indicates that SFN can ameliorate cardiac damage caused by Ang II by activation of Nrf2 signalling, confirmed by using wild‐type as well as knockout of Nrf2 gene mice.^8^ Interestingly, SFN treatment results in a persistently high expression of Nrf2 in the heart for another 3 more months after withdrawing SFN.^8^ However, it is still unclear about the underlying mechanism.

Accumulating studies suggest that SFN, as a potent activator of Nrf2, exhibits the antioxidant ability by up‐regulating Nrf2‐mediated cytoprotective genes expression through modification of Keap1 cysteine residues, activation of MAPK, phosphatidylinositol 3‐kinase (PI3K) and protein kinase C (PKC) pathways, which results in the phosphorylation, nuclear accumulation and increased transcription and stability of Nrf2.^37^, ^38^ Notably, SFN can also regulate the Nrf2 expression by epigenetic modification, including DNA methylation and histone modification.^39^, ^40^ In prostate cancer, the transcription suppression of Nrf2 is closely correlated to enhanced CpGs methylation level in the Nrf2 promoter region, whereas SFN exerts a chemopreventive effect by restoring Nrf2 through demethylation of CpGs and the accumulation of histone H3 acetylation at the Nrf2 promoter, achieved by the inhibition of DNA methyltransferases (DNMTs) and histone deacetylases (HDACs).^39^ It was also observed that SFN activates Nrf2 by down‐regulation of CpG methylation in mouse skin epidermal JB6 (JB6 P+) cells.^41^ Based on the above studies, we are interested in exploring whether SFN can activate cardiac Nrf2 expression though an epigenetic mechanism, thereby preventing Ang II‐induced cardiac damage. This study will provide new research direction and strategy for preventing Ang II‐induced cardiac damage.

## 2 MATERIALS AND METHODS

### 2.1 Animals

Male C57/BL mice at the age of 8 weeks were purchased from Beijing Experimental Animal Technical Co. LTD. The mice were housed in the Animal Center of Jilin University (Changchun, China). All animal procedures were approved by the Animal Care and Use Committee of the Chinese Academy of Medical Sciences (Beijing, China). The mice were randomly divided into four groups (n = 5‐7 each group), namely control group, SFN group, Ang II group and Ang II plus SFN (Ang II/SFN) group according to our previous method.^8^ Ang II injury and SFN treatment animal models were established based on our previous study.^10^, ^42^ Briefly, mice were injected subcutaneously with Ang II (NUC201, Sigma‐Aldrich) at a dose of 0.5 mg/kg every other day for 2 months. SFN (0.5 mg/kg, NUC201, Sigma‐Aldrich) was subcutaneously administered for 5 days a week for 3 months. The control group was given the same dose of SFN formulation solvent. At the time point of SFN treatment for 3 months, half of the mice in each group were sacrificed, and the remaining mice were not treated with SFN and kept for another 3 months, and then sacrificed for experimental measurements.

Dose of SFN (0.5 mg/kg) was selected based on our and other publications.^8^, ^43^ According to the method of converting equivalent doses from animal to human based on the body surface area guided by the FDA, 0.5 mg/kg SFN in mice is converted to a human dose of 0.0405 mg/kg. 300 grams of broccoli contains about 2.63 mg of SFN, which may be higher after a short time of cooking.^44^, ^45^ Moreover, in some clinical studies, the dose of SFN used to treat chronic diseases is usually higher than the dose of 0.5 mg/kg per day used in this study.^46^, ^47^ Therefore, the dose of SFN used in this study is relatively low and safe.

### 2.2 Echocardiography

Echocardiography (Echo) was performed to measure heart function for mice anaesthetized with Avertin (NUC201, Sigma‐Aldrich) by using a high‐resolution imaging system suitable for small animals (Vevo 770, Visual Sonics), equipped with a high‐frequency ultrasound probe (RMV‐707B). Echo analyses include chamber dimensions and cardiac function.

### 2.3 Preparation of protein lysates and western blotting

A high‐speed tissue homogenizer was used to lyse heart tissues in ice‐cold 1× RIPA buffer (Cell Signaling Technology) supplemented with protein inhibitors (NUC201, Sigma‐Aldrich). Use 4% to 15% SDS‐polyacrylamide gel electrophoresis (SDS‐PAGE) to separate proteins and transfer them to PVDF membrane (Millipore). After blocking with 5% BSA, incubate the membrane with specific primary antibodies from Santa Cruz Biotechnology at 4°C overnight, including CAT (1:500), HO‐1 (1:500), CTGF (1:1000), 3‐NT (1:1000), 4‐HNE (1:1000), ICAM‐1 (1:500), IL‐1β (1:500), DNMT3a and 3b (1:1000 each), HDAC2, 3, and 5 (1:1000 each), and β‐actin (1:1000), then incubate with HRP‐conjugated secondary antibodies (1:2000, Santa Cruz Biotechnology) for 1 hour at room temperature. The antibody‐bound proteins were detected with a Super Signal enhanced chemiluminescence (ECL) detection and Gel Documentation 2000 system (Bio‐Rad). Use Image J software to analyse the densitometry of the bands.

### 2.4 RNA isolation and quantitative real‐time polymerase chain reaction (qPCR)

The total RNA was extracted from the hearts of mice using Trizol reagent (Invitrogen). According to the manufacturer's instructions, first‐strand cDNA was synthesized from total RNA using the SuperScript III First‐Strand Synthesis System (Invitrogen). The mRNA expression level was qualified using the first‐strand cDNA as a template by quantitative real‐time PCR (qPCR) with Power SYBR Green PCR Master Mix (Applied Biosystems), and GAPDH was used as an internal loading control. The following primer sequences for Nrf2, HO‐1, CAT, GAPDH were used: Nrf2, 5′‐TCACACGAGATGAGCTTAGGGCAA‐3′ (sense) and 5′‐TACAGTTCTGGGCGGCGACTTTAT‐3′ (antisense); HO‐1, 5′‐CCTCACTGGCAGGAAATCATC‐3′ (sense) and 5′‐CCTCGTGGAGACGCTTTACATA‐3′ (antisense); CAT, 5′‐GGAGGCGGGAACCCAATAG‐3′ (sense) and 5′‐GTGTGCCATCTCGTCAGTGAA‐3′ (antisense); GAPDH, 5′‐TCAACAGCAACTCCCACTCTTCCA‐3′ (sense) and 5′‐ACCCTGTTGCTGTAGCCGTATTCA‐3′ (antisense).

### 2.5 Heart to body weight ratio measurement and histology

After the mice were weighed and sacrificed, the hearts were taken out and weighed. Then calculate the ratio of heart to body weight. The heart tissues were fixed in 10% formalin, embedded in paraffin and cut into 5 µm thickness. The heart sections were deparaffinized and rehydrated in a graded alcohol series, then subjected to Masson's Trichrome staining for collagen deposition.

### 2.6 Bisulfite genomic sequencing (BGS)

Genomic DNA was extracted from the heart tissues of mice using a QIAamp DNA Mini kit (Qiagen). Then, bisulfite was used to convert DNA with an EZ DNA Methylation‐Gold Kit (Zymo Research Corp.). TA cloning was performed to amplify the converted DNA using Platinum Taq DNA Polymerase (Invitrogen) with primers that amplify the first 15 CpGs located between −1226 and −863 of the murine Nrf2 gene with the translation initiation site (TIS) defined as position 1.^48^ The primer sequences were 5′‐AGTTATGAAGTAGTAGTAAAAA‐3′ (sense) and 5′‐ACCCCAAAAAAATAAATAAATC‐3′ (antisense). The PCR products were cloned into a PCR4 TOPO vector. The QIAprep Spin Miniprep kit (Qiagen) was used to amplify and purify a plasmid containing PCR products of 10 colonies from each treatment group, and then sequenced (GeneWiz).

### 2.7 DNMT/HDAC activity assay

Total DNMT/HDAC activity was assayed with a fluorometric EpiQuick™ DNMT/HDAC Activity/Inhibition Assay kit (Epigentek). The fluorescence density was read at 405 nm by using a Tecan microplate reader plate reader.

### 2.8 Chromatin Immunoprecipitation (ChIP) assay

Chromatin immunoprecipitation (ChIP) assay was performed following the manufacturer's protocol. In short, heart tissues were cross‐linked with formaldehyde to a final concentration of 1% at room temperature for 15 min, then add 2.5 mol/L glycine to formaldehyde and incubate for 10 min. After washing twice with PBS, resuspend the tissues in Lysis Buffer containing a mixture of protease inhibitors, lyse with a homogenizer and sonicate to produce the 200‐500 bp DNA fragments in ice‐cold water. The samples were centrifuged at 13 500 *g*. Using a dilution buffer to dilute the chromatin solutions, 10 μL of each sample was used as input control. Diluted chromatin solutions were then immunoprecipitated with protein A magnetic beads and anti‐acetyl‐Histone 3 (Ac‐H3; Cell Signaling Technology) antibody or non‐specific Rabbit IgG. The solutions were then slowly shaken overnight at 4°C. Then a magnetic separator was used to collect the immunoprecipitated complex‐magnetic beads. The purified DNA solution was used for regular PCR amplification using the primers: 5′‐TGAGATATTTTGCACATCCGATA‐3′ (sense) and 5′‐ACTCTCAGGGTTCCTTTACACG‐3′ (antisense), which covers the DNA sequence of the first 15 CpGs of murine Nrf2.

### 2.9 Statistical analysis

Data are represented as means ± SD values (n = 5‐7 per group). The data analyses were performed using two‐way analysis of variance (ANOVA) or Tukey's test with Origin 7.5 software (OriginLab Corporation). Differences were considered statistically significant when *P* < .05.

## 3 RESULTS

### 3.1 SFN improves Ang II‐induced cardiac remodelling and dysfunction in mice

**FIGURE 1:**
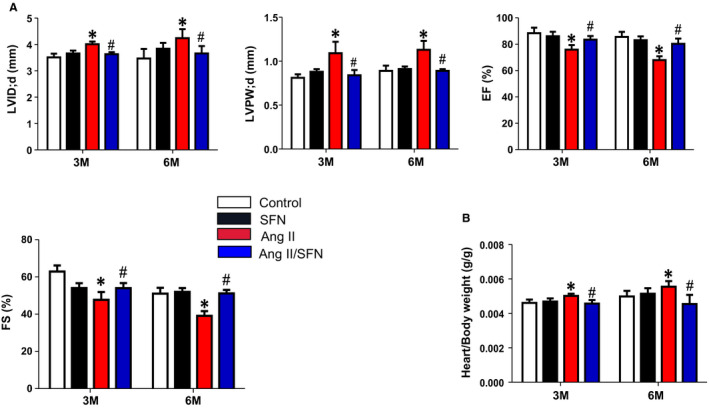
SFN improves Ang II‐induced cardiac remodelling and dysfunction in mice. Wild‐type mice were injected subcutaneously with Ang II (0.5 mg/kg) every other day for 2 months, with or without SFN (0.5 mg/kg body weight) 5 days per week for 3 months and then kept until 6 months. A, Cardiac function was measured by Echo. B, The ratios of heart weight to body weight were calculated. Data were presented as the mean ± standard deviation (SD, n = 5‐7). *P < .05 vs control; ^#^ *P* < .05 vs Ang II. FS, fractional shortening; EF, ejection fraction;LVID; d, Left ventricular end‐diastolic diameter; LVPW; d, Left ventricular end‐diastolic posterior wall thickness

To confirm the protective effect of SFN for cardiac dysfunction caused by Ang II, Echo was performed to detect cardiac function in mice. We found that 2‐month Ang II administration caused cardiac remodelling and dysfunction, defined by enhanced LVID and LVPW, and reduced EF and FS (Figure 1A). The cardiac hypertrophy was also manifested as increased ratios of heart to body weight in the Ang II group (Figure 1B). However, all these cardiac pathological alterations were found to be attenuated by 3‐month SFN treatment (Figure 1A,B).

### 3.2 SFN inhibits Ang II‐induced cardiac inflammatory factor expression, oxidative damage and fibrosis

**FIGURE 2:**
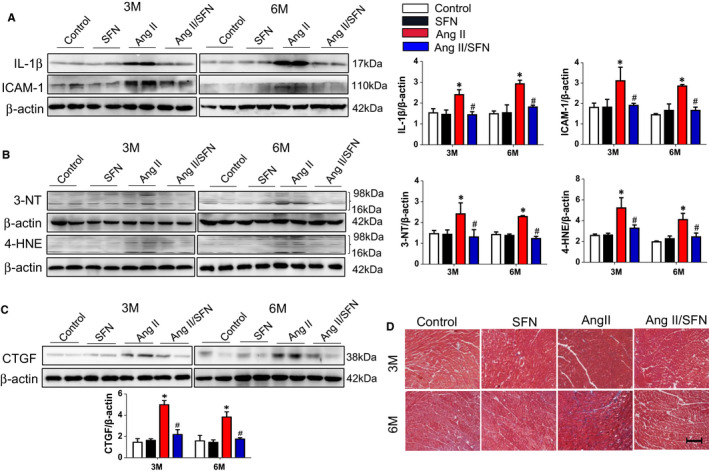
SFN inhibits cardiac inflammatory factors expression, oxidative damage and fibrosis induced by Ang II. Western blot analysis includes the expression of inflammatory factors ICAM‐1 and IL‐1β (A), oxidative stress markers (3‐nitrotyrosine (3‐NT) and 4‐hydroxy‐2‐nonenal (4‐HNE) (B), and the cardiac fibrotic marker CTGF and quantitative analysis (C). D, Masson staining was applied to determine the deposition of collagen (collagen fibres are blue, scale bar = 0.5 cm). Data were presented as the mean ± SD (n = 5‐7). *P < .05 vs control; ^#^ *P* < .05 vs Ang II

It has been confirmed that the inflammatory response and oxidative stress involve the pathogenesis of cardiac damage caused by Ang II.^12^ The 2‐month Ang II injection resulted in a significantly increased expression of ICAM‐1 and interleukin‐1β (IL‐1β) in cardiac tissues, which were dramatically decreased after SFN treatment (Figure 2A). In response to Ang II treatment, the expression of 3‐NT and 4‐HNE (oxidative stress markers) was highly induced, but almost completely inhibited by 3‐month SFN treatment (Figure 2B).

Reportedly cardiac fibrosis, remodelling and dysfunction is often the ultimate consequence of cardiac inflammation and oxidative damage caused by Ang II.^6^ Cardiac fibrosis is referred to the elevated protein expression of the fibrotic marker CTGF (connective tissue growth factor) as well as collagen deposition.^8^ We observed that expression of cardiac CTGF was remarkably higher in the Ang II group (Figure 2C). Moreover, Ang II induced the deposition of collagen in the mouse heart tissues, especially more evident at 6 months by Masson staining, while SFN treatment eliminated the cardiac fibrosis response (Figure 2D). These results suggested that administration of SFN could effectively ameliorate cardiac damage induced by Ang II.

### 3.3 SFN up‐regulates the expression of Nrf2 and its downstream antioxidant genes

**FIGURE 3:**
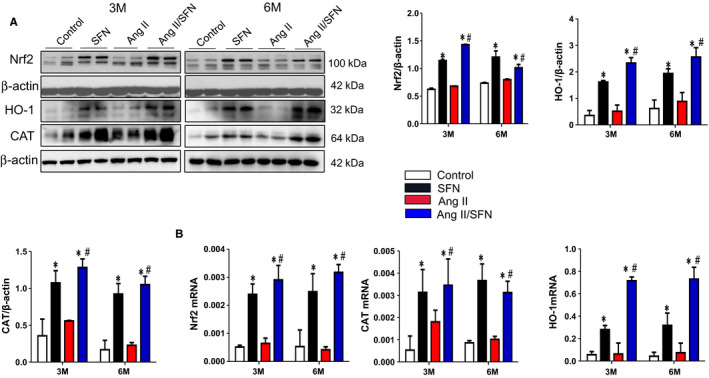
SFN up‐regulates the expression of Nrf2 and its downstream antioxidant genes. The expression of Nrf2 and its downstream genes of haem oxygenase 1 (HO‐1) and catalase (CAT) was examined by western blot (A), and the transcription of Nrf2, CAT and HO‐1 was quantified by qPCR (B), along with quantitative analysis. Data were presented as the mean ± SD (n = 5‐7). *P < .05 vs control; ^#^ *P* < .05 vs Ang II

To confirm the indispensable role of Nrf2 in the SFN‐mediated prevention of cardiac damage induced by Ang II, we next detected the transcription capacity of Nrf2, demonstrated by CAT and HO‐1 expression. Compared to the control group, Ang II injection did not cause a obvious difference in the transcriptional and protein expression of Nrf2, CAT and HO‐1, but SFN treatment significantly increased the Nrf2 expression and its downstream antioxidant genes expression, which was much higher in the Ang II/SFN group than that in the Ang II group (Figure 3A,B). These results indicate that SFN treatment significantly up‐regulated the expression and function of cardiac Nrf2, which possibly mediated the cardioprotective effect of SFN in Ang II‐stimulated mice.

### 3.4 SFN activates cardiac Nrf2 partially through inhibiting CPG island methylation in the Nrf2 promoter

**FIGURE 4:**
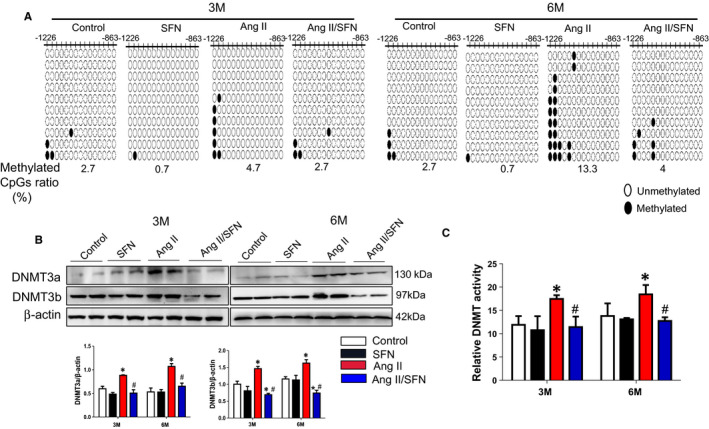
SFN activate cardiac Nrf2 partially through inhibiting CPG islands methylation in the Nrf2 promoter. A, Bisulfite genomic sequencing was applied to assess the CpGs methylation status in the promoter of Nrf2 gene. Black dots and open circles presented methylated CpGs and non‐methylated CpGs, respectively. B, The expression of DNMT3a and 3b was detected by western blot. C, The DNMT activity kit was used to examine the total activity of DNMT. Data were presented as the mean ± SD (n = 5‐7). *P < .05 vs control; ^#^ *P* < .05 vs Ang II

Transcription activation of Nrf2 is inhibited due to the hypermethylation of the first 15 CpGs at the Nrf2 gene promoter in a TRAMP mouse model and JB6 P+ cells.^39^, ^41^ Therefore, bisulfite sequencing was performed to explore whether SFN could promote the CpGs demethylation at the Nrf2 promoter, located between −863 and −1226. As shown in Figure 4A, compared to the control group, Ang II promoted the hypermethylation of the 4.7% CpGs at 3 months, while the methylation level was reduced to 2.7% by SFN treatment (Figure 4A). At 6 months, the methylation rate after Ang II stimulation was 13.3%, particularly higher than that at 3 months, and treatment with SFN lowered the methylation rate to 4% (Figure 4A).

DNA methylation is mediated by DNMTs, mainly including DNMT1 (maintenance methyltransferase) and DNMT3a and 3b (de novo methyltransferases). We next examined the expression and activity changes of DNMTs in the cardiac tissues of mice. Ang II treatment greatly increased the protein expression of DNMT3a and 3b and the global DNMT activity (Figure 4B,C), which were reduced by SFN treatment. The above results show that SFN may reduce CpG methylation ratios of the Nrf2 promoter by inhibition of DNMTs.

### 3.5 SFN activates cardiac Nrf2 partially through enhancing the acetylation of histone H3 in the Nrf2 promoter

**FIGURE 5:**
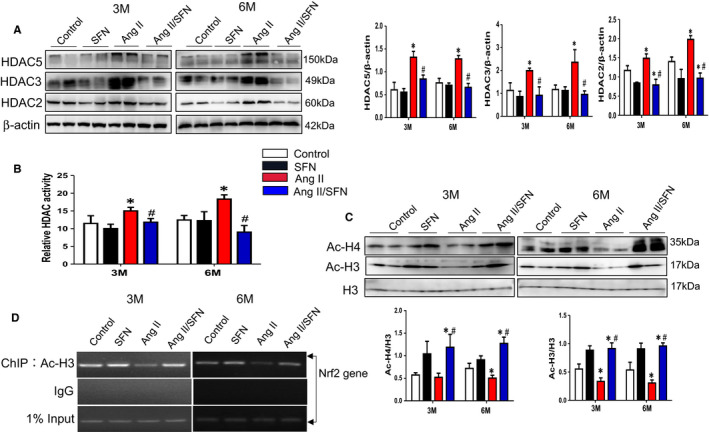
SFN activates cardiac Nrf2 partially through enhancing the acetylation of histone H3 in the Nrf2 promoter. A, The expression of HDAC2, 3 and 5 was examined by western blot. B, The total activity of HDAC was detected by HDAC activity kit. C, Western blot was performed to examine the expression of Ac‐H3 and Ac‐H4. D, ChIP assay was used to detect the Ac‐H3 enrichment in Nrf2 promoter region. Data were presented as the mean ± SD (n = 5‐7). *P < .05 vs control; ^#^ *P* < .05 vs Ang II

Histone modification is also an important epigenetic modification, and numerous reports have shown that SFN is an inhibitor of HDACs.^49^ Ang II caused a dramatically elevated protein levels of HDACs (HDAC2, 3, and 5) compared with the control group, while almost eliminated to the normal level by SFN treatment at 3 and 6 months (Figure 5A). Correspondingly, SFN inhibited the activity of global HDACs highly induced by Ang II (Figure 5B). The inhibition of HDAC accounts for the enhancement of histone H3 and H4 acetylation, which will result in chromatin remodelling and facilitate gene transcription. Our results show that, compared with the control group, the global level of acetylated H3 and H4 (Ac‐H3 and Ac‐H4, active chromatin markers) was decreased in the Ang II group, but they were strongly increased after SFN treatment (Figure 5C). Next, the ChIP analysis was further used to explore the effect of SFN on the Ac‐H3 enrichment in the Nrf2 promoter. Consistent with the expression change of global Ac‐H3, Ang II caused a decreased accumulation of Ac‐H3 in the Nrf2 promoter region, whereas SFN treatment recovered the enrichment of Ac‐H3 at 3 and 6 months (Figure 5D). These results indicate that SFN promoted the accumulation of Ac‐H3 in the Nrf2 promoter region via the inhibition of HDACs.

## 4 DISCUSSION

Our previous study has demonstrated that SFN exhibits its preventive effect for Ang II‐induced cardiomyopathy via activating Nrf2.^8^ Furthermore, SFN treatment induced a persistent activation of Nrf2 for at least 3 months afterwards.^8^ However, no defined mechanisms can clarify the long‐term regulatory effect of Nrf2 by SFN. We find that (a) SFN can prevent cardiac damage caused by Ang II, which is related to the up‐regulation of Nrf2, (b) under Ang II treatment, SFN inhibits the expression and activity of DNMTs and HDACs, thereby reducing the methylation rate of CpGs and promoting the Ac‐H3 accumulation in the Nrf2 promoter region, and to a certain extent, enhances the transcription of Nrf2 in the heart. Therefore, our research suggests for the first time that SFN has the potential to activate cardiac Nrf2 through epigenetic modification.

Oxidative stress is a key contributor to cardiomyopathy induced by Ang II.^11^ In our study, chronic Ang II stimulation caused a considerable increase in cardiac inflammatory factors, oxidative damage and fibrosis, ultimately leading to cardiac remodelling and dysfunction (Figures 1 and 2). SFN has received extensive attention as a phytochemical with multiple bioactivities, including antioxidative, anti‐inflammatory and anticancer, and many commercial SFN supplements are also available.^26^, ^28^ Additionally, several clinical trials have evaluated the safety of SFN at the doses employed. Firstly, two randomized, placebo‐controlled human studies have investigated the effects of SFN in vivo, the results indicate that broccoli sprout extracts containing SFN are well tolerated and cause no significant adverse events when administered orally by healthy volunteers at a dose of 15 µmol/L for 7 days.^28^, ^29^ The first clinical trial in men with recurrent prostate cancer also confirms the safety of SFN‐rich extracts (200 µmol/day).^50^ Our results showed that SFN inhibited Ang II‐induced cardiac hypertrophy (Figure 1), inflammatory factor expression (Figure 2A), oxidative stress (Figure 2B) and fibrosis (Figure 2C‐D), and significantly improved cardiac dysfunction (Figure 1A). These results demonstrate that SFN can effectively prevent chronic cardiac damage caused by Ang II, even persisting for 3 months after the cessation of SFN, which is in line with our previous findings.^8^

Nrf2, as a transcription factor, enhances the capacity of endogenous antioxidant defence against oxidative damage.^22^ The activation of Nrf2 has been shown to suppress oxidative stress‐related cardiac hypertrophy,^25^, ^51^ while exacerbated by knockdown of Nrf2.^23^ Therefore, Nrf2 is widely regarded to be activated under stress, however, our study suggested that the expression and transcription function of Nrf2 was not significantly increased after 2 months of Ang II stimulation (Figure 3). It is possible that the activation of some inhibitory factors or long‐term oxidative stress stimulation has impaired Nrf2 function, causing the disappearance of its compensatory protective effect, which will ultimately result in more severe cardiac damage. In addition, numerous studies show that SFN functions as indirect antioxidants and prevent heart disease caused by oxidative stress through activating Nrf2.^10^, ^33^, ^34^, ^51^, ^52^ Also, our current study indicated that SFN enhanced the expression and function of Nrf2, manifested as increased transcription of Nrf2 and its mediated antioxidant genes, including CAT and HO‐1(Figure 3). Combined with our previous work, the cardioprotective effect of SFN is known to be correlated with activation of Nrf2.^8^

Growing evidence suggests that natural phytochemicals such as SFN, curcumin, angelica and tocopherol can reactivate the silenced Nrf2 gene by epigenetic modification, including DNA methylation and histone modification.^39^, ^41^, ^53^, ^54^, ^55^, ^56^ SFN has been shown to be an inhibitor of DNMTs and HDACs.^56^ In the study of prostate TRAMP mice, inactivation of Nrf2 may be attributed to the hypermethylation of CpG islands, while SFN can reactivate Nrf2 via DNA demethylation, accompanied by an inhibition of DNMTs and HDACs.^39^ Additionally, SFN treatment raises the expression of Nrf2 and its target genes through a similar epigenetic mechanism, thereby significantly inhibiting TPA‐induced malignant transformation of JB6 P+ cells.^41^ In prostate epithelial and breast cancer cells, SFN exerts its anticancer effect by inhibiting HDAC activity.^56^ SFN administration in mice inhibits HDACs in the colonic mucosa, following by increased acetylation of H4 and H3(Ac‐H4 and Ac‐H3).^57^ In TRAMP mice with prostate cancer, SFN treatment increased Ac‐H3 in the Nrf2 promoter region, thus facilitating the expression of Nrf2 and NQO1.^39^

To elucidate the mechanism accounting for the lasting activation of Nrf2 by SFN in the hearts of mice, we detected the CpGs methylation status located in the Nrf2 promoter region by bisulfite sequencing. To a large extent, these CpGs determine the transcription of Nrf2.^58^ This study indicates that Ang II promoted the CpGs hypermethylation of the Nrf2 promoter region, especially at 6 months, whereas SFN reduced the methylation ratios (Figure 4A). Additionally, Ang II caused an increase in total DNMT and HDAC activity, and becomes significantly decreased after SFN administration (Figures 4C and 5B). Meanwhile, Ang II enhanced the protein level of DNMTs (DNMT3a and 3b), as well as HDACs (including HDAC2, HDAC3 and HDAC5), but were significantly inhibited by SFN (Figures 4B and 5A). With respect to histone acetylation, SFN treatment significantly increased Ac‐H3/H4 (active chromatin markers) expression (Figure 5C). Specifically, SFN treatment promoted the enrichment of Ac‐H3 at the Nrf2 promoter through the ChIP assay, compared to Ang II stimulation (Figure 5D).

While DNA methylation and histone acetylation can independently regulate gene expression, several studies indicate that they may interact with each other to establish and maintain different chromatin states.^59^, ^60^, ^61^ Early studies have shown that the interaction between DNA methylation and histone deacetylation is mediated by a group of proteins with methyl DNA binding activity, including methyl CpG binding protein 2 (MeCP2), methyl CpG binding domain protein 1 (MBD1).^62^ These proteins localize to DNA methylated promoters and recruit histone deacetylases (HDACs) and other co‐repressors to form a transcription repressor complex. This complex binds to the promoter region of specific DNA sequences, thereby inhibiting gene expression.^63^, ^64^, ^65^ Overall, DNA methylation and histone deacetylation are considered to inhibit gene transcription with a synergistic effect.^66^, ^67^ In our current study, SFN may impede the formation of transcription repressor complexes, and the release of these complexes further elevates the Ac‐H3 enrichment in the Nrf2 promoter, at least partially contributing to reactivation of Nrf2.

Under basic conditions, Keap1, a negative regulator of Nrf2, mediates the ubiquitination and proteasome degradation of Nrf2.^68^ It is reported that epigenetic modifications at the Keap1 promoter are involved in the modulation of Nrf2‐mediated antioxidant genes.^69^ The Keap1 promoter hypermethylation mainly exhibits protective effects for oxidative stress‐related diseases, followed by decreased Keap1 and increased Nrf2 expression that may counteract the reduced transcription of Nrf2 due to its promoter hypermethylation.^69^ This may explain why Nrf2 expression was not reduced after Ang II administration compared to the control mice (Figure 3), but Nrf2 promoter methylation and epigenetic modification enzymes were significantly increased (Figures 4 and 5). It is worth noting that SFN had no strong impact on the level of Ac‐H3 at the Nrf2 promoter and the epigenetic modifying enzymes (DNMTs/HDACs) expression under the basal condition, but up‐regulated Nrf2‐mediated antioxidative gene expression (Figures 3, 4 and 5), suggesting that the basal Nrf2 level in the heart is probably not modulated via epigenetic modification. Therefore, other potential mechanisms may involve the activation of Nrf2.

The modulation mechanism of Nrf2 signalling pathway can be roughly summarized as Keap1‐dependent and Keap1‐independent manners. The direct modification of Keap1 cysteine residues by SFN is considered as the Keap1‐dependent model, which inhibits Nrf2 polyubiquitination and degradation, translocating Nrf2 to nucleus.^37^, ^70^ Nrf2 can also be regulated independently of Keap1. Evidence indicates that SFN may indirectly activate Nrf2 by affecting the activity of several upstream kinases (including MAPK, PI3K and PKC), these intracellular kinases phosphorylate Nrf2, alter nuclear‐cytoplasmic trafficking of Nrf2 or regulate Nrf2 protein stability.^38^, ^70^ Additionally, our previous study also found that SFN activates Nrf2 through protein kinase B (AKT) signalling pathways.^8^ The study in rat cardiomyocytes revealed that SFN has an ability to activate ERK,^71^ and then triggers Nrf2 phosphorylation and nuclear translocation, resulting in a cytoprotective effect against oxidative damage. However, further investigation is required to determine the exact mechanism.

## 5 CONCLUSION

**FIGURE 6:**
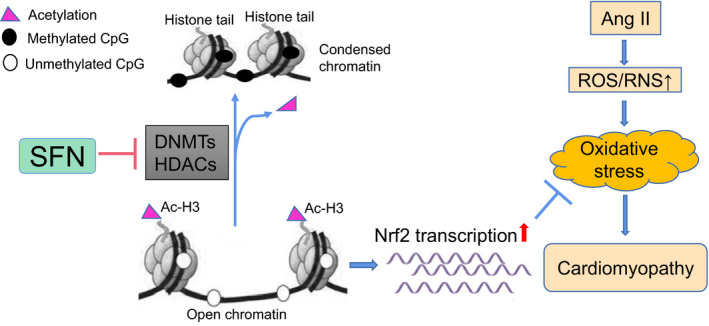
SFN reduces the CpGs methylation and promotes the enrich of Ac‐H3 in Nrf2 promoter by inhibiting DNMTs and HDACs, partially contributing to the long‐acting activation of cardiac Nrf2, thereby preventing Ang II‐induced cardiomyopathy

Current research indicates that SFN can protect against cardiac damage induced Ang II through up‐regulation of Nrf2 and its downstream antioxidative genes CAT, HO‐1. Furthermore, SFN can activate the Nrf2 gene under Ang II treatment through epigenetic modification, including DNA methylation and histone acetylation, by reducing the methylation level and enhancing the Ac‐H3 accumulation in the Nrf2 promoter region through the inhibition of DNMTs and HDACs. The epigenetic mechanism can partially contribute to the persistent long‐acting activation of Nrf2 by SFN in the heart (Figure 6).

## CONFLICT OF INTEREST

The authors report no conflicts of interest in this work.

## AUTHOR CONTRIBUTION

**Xuling Su:** Data curation (equal); Formal analysis (equal); Methodology (equal); Writing‐original draft (lead). **Shudong Wang:** Investigation (equal); Methodology (equal); Software (equal). **Haiying Zhang:** Data curation (equal); Investigation (equal); Methodology (equal); Software (equal). **Ge Yang:** Data curation (equal); Methodology (equal); Software (equal). **Yang Bai:** Data curation (equal); Investigation (equal); Methodology (equal); Software (equal); Visualization (equal). **Pinyi Liu:** Data curation (equal); Formal analysis (equal); Methodology (equal); Writing‐original draft (equal). **Lingbin Meng:** Conceptualization (equal); Investigation (equal); Project administration (equal); Writing‐review & editing (equal). **Xin Jiang:** Conceptualization (equal); Project administration (equal); Writing‐review & editing (equal). **Ying Xin:** Funding acquisition (equal); Project administration (equal); Supervision (equal); Visualization (equal); Writing‐review & editing (equal).

## Data Availability

The data used to support the findings of this study are included within the article.
